# Systemic *Candida parapsilosis* Infection Model in Immunosuppressed ICR Mice and Assessing the Antifungal Efficiency of Fluconazole

**DOI:** 10.1155/2015/370641

**Published:** 2015-07-09

**Authors:** Yu'e Wu, Fangui Min, Jinchun Pan, Jing Wang, Wen Yuan, Yu Zhang, Ren Huang, Lixin Zhang

**Affiliations:** ^1^Guangdong Laboratory Animals Monitoring Institute, Guangdong Provincial Key Laboratory of Laboratory Animals, Guangzhou 510663, China; ^2^Institute of Microbiology, Chinese Academy of Sciences, Beijing 100080, China

## Abstract

This study was to establish a systemic *C. parapsilosis* infection model in immunosuppressed ICR mice induced by cyclophosphamide and evaluate the antifungal efficiency of fluconazole. Three experiments were set to confirm the optimal infectious dose of *C. parapsilosis*, outcomes of infectious model, and antifungal efficiency of fluconazole in vivo, respectively. In the first experiment, comparisons of survival proportions between different infectious doses treated groups showed that the optimal inoculum for *C. parapsilosis* was 0.9 × 10^5^ CFU per mouse. The following experiment was set to observe the outcomes of infection at a dose of 0.9 × 10^5^ CFU *C. parapsilosis*. Postmortem and histopathological examinations presented fugal-specific lesions in multiorgans, especially in kidneys, characterized by inflammation, numerous microabscesses, and fungal infiltration. The CFU counts were consistent with the histopathological changes in tissues. Th1/Th2 cytokine imbalance was observed with increases of proinflammatory cytokines and no responses of anti-inflammatory cytokines in sera and kidneys. In the last experiment, model based evaluation of fluconazole indicated that there were ideal antifungal activities for fluconazole at dosages of 10–50 mg/kg/d. Data demonstrates that the research team has established a systemic *C. parapsilosis* infection model in immunosuppressed ICR mice, affording opportunities for increasing our understanding of fungal pathogenesis and treatment.

## 1. Introduction

*Candida*, usually kept as harmless commensals in healthy individuals, may become opportunistic pathogens in susceptible hosts, especially in severely drug-immunosuppressed or immunodeficient patients [1–3]. During the past two decades, the candidemia causative agent has changed from* C. albicans *to non-*C. albicans*, and the patients infected with the non-*C. albicans* were gradually increased.* Candida* infections have accounted for about 8 to 9 percent of hospital-acquired infections and become the fourth most common cause of such infections [4, 5].* C. parapsilosis, *a typical commensal of human skin, has emerged notoriously for its capacity to grow in total parenteral nutrition and to form biofilms on catheters and other implanted devices [6, 7]. During the last decade, the incidence of* C. parapsilosis* has dramatically increased and become the second most commonly isolated* Candida* species from blood cultures.

Given the incidence of disease and the unacceptably high mortality associated with* C. parapsilosis*, there is an urgent need of more effective preventive, diagnostic, and therapeutic strategies. Experimental animal models are a critical component of understanding the pathogenesis and host resistance to infection and to development of more efficacious antifungal therapies. Previously, an immunocompromised mouse model of* C. parapsilosis *established by us has been used to evaluate microbial metabolites as combination agents for the treatment of fungal infections [8]. After further optimization for establishment procedures, the animal model was more stable and presented outcomes of systemic infection. The present paper will describe in detail the outcomes of the systemic mouse* C. parapsilosis* model, including mortality, tissues fungal burdens, histopathology, serum and renal cytokines, and the usage of the model for evaluation of fluconazole.

## 2. Materials and Methods

### 2.1. Animals and Ethics Statement

SPF female ICR mice aged from 4 to 6 weeks and weighed 20 to 22 g were used in this study. Animals were purchased from SLAC Laboratory Animal Centre Co., Shanghai, and had never been used for any experimental procedures previously. After arrival, animals were acclimated for 3 days before the experiments.

Animal use protocols were reviewed and approved by IACUC of Guangdong Laboratory Animal Monitoring Institute in accordance with the* Guide for the Care and Use of Laboratory Animals *[9]. Animals were bred in negative pressure isolation cages in an animal negative pressure facility with an approval of and oversight by the Local Provincial Institutional Environmental Health and Safety Office.

### 2.2. Fungal Strain and Inoculum Preparation

*C. parapsilosis* ATCC22019 normally stored at −86°C was used in this study. Stock inoculum suspensions of* C. parapsilosis* were obtained from >20 h cultures in RPMI medium 1640 incubated at 30°C with shaking at 150 rpm. And >95% of* C. parapsilosis* cells should be blastoconidia by microscopic examination.

### 2.3. Cyclophosphamide Induced Immunosuppression

Ten mice were intraperitoneally injected with cyclophosphamide (CY) (100 mg/kg weight/d) for continuous 3 days. Blood samples (0.20 mL) were collected via fossa orbitalis vein daily from 0 to 6 days after the injection of CY. Then, total leukocyte counts were performed by the* Sysmex XT-2000iv* automatic hematology analyzer. Another 10 mice receiving normal saline were set as controls.

### 2.4. Study Design

The first experiment was set to confirm the infectious dose. The immunosuppressed mice receiving CY were inoculated with 0.1 mL* C. parapsilosis* suspension via tail vein at doses of 1 × 10^2^ CFU, 0.9 × 10^5^ CFU, 5.0 × 10^5^ CFU, and 8 × 10^5^ CFU, respectively. The control mice receiving normal saline were inoculated with 0.1 mL normal saline via tail vein. According to the results of survival curves, the optimal infectious dose would be confirmed.

In the second experiment, another immunosuppressed group inoculated with optimal infectious dose of* C. parapsilosis *was set to observe the outcomes of infection. At days 1, 4, and 6 postinfection, 3 mice were euthanized, sera were collected, and target organs (heart, liver, spleen, lung, kidney, and brain) were excised for tissue fungal burdens, pathological examination, and cytokine measurement.

The third experiment was designed to evaluate fluconazole (Sigma). Four groups of infected mice were treated intraperitoneally with fluconazole at the dosages of 0, 0.5, 10, and 50 mg/kg/d of body weight 1 h postinfection for 7 consecutive days. Survival rates and tissue fungal burdens were used to evaluate the antifungal efficacy of fluconazole.

### 2.5. Clinical Assessment

Animals were observed daily throughout the study for alterations in behavior, appetite, and mortality.

### 2.6. Postmortem and Histopathological Examinations

The mice were sacrificed by CO_2_ inhalation. Heart, liver, spleen, lung, kidney, brain, stomach, and bladder were removed immediately and preserved in 10% formalin. After paraffin embedding and sectioning, standard 5 *μ*m sections were cut and stained with hematoxylin and eosin (H&E) and periodic acid-Schiff (PAS).

### 2.7. Tissue Fungal Burdens

Half of the target organs were homogenized in 1.0 mL of sterile normal saline. Tissue homogenates from individual mice were serially diluted on SDA plates and incubated for 48 h at 35°C. Results are expressed as CFU log_10_ per organ.

### 2.8. Cytokine Measurement

IL-1*α*, IL-1*β*, IL-2, IL-4, IL-5, IL-6, IL-10, IL-17, IL-23, GM-CSF, IFN-*γ*, and TNF-*α* in serial sera and renal samples of control and infected mice were measured by cytometric bead array.

### 2.9. Data Analysis

All data was expressed as mean ± SD. Between-group differences of quantitative data were analyzed by *t*-tests. While the between-group differences of survival cures were performed by* Log-rank (Mantel-Cox) Test*. Significance was judged at the 0.05 level.

## 3. Results

### 3.1. CY Induced Immunosuppression

**Figure 1:**
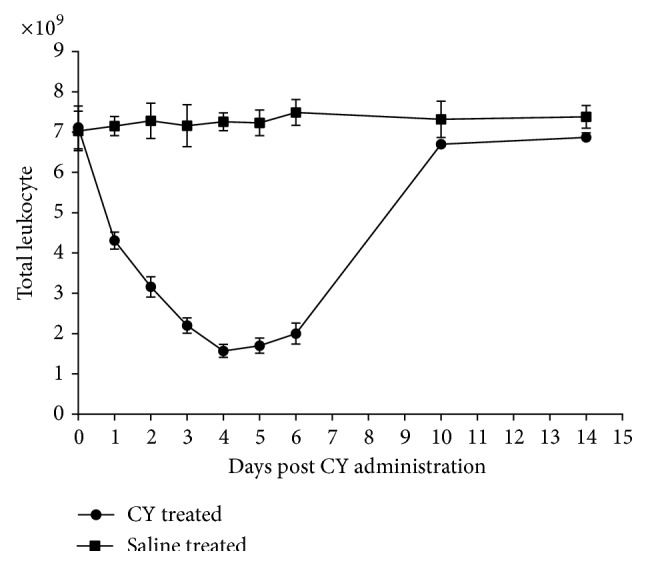
Total leukocyte counts. A time corresponding decrease was found in CY treated mice from days 1 to 4. The lowest data emerged at day 4 (*n* = 10).

No significant changes were found in total leukocyte counts for the control mice injected with saline. However, the mice receiving CY showed a time corresponding decrease from days 1 to 4. At day 4, the total leukocyte counts of mice receiving CY reached the lowest levels followed by a gradual increase to basal levels till day 14 (Figure 1). The results indicate that the optimal infection time points are days 3 to 6 after CY administration.

### 3.2. Confirmation of Infectious Dose by Survival Proportions

In the first study, every immunosuppressed group presented death of animals postinfection. All except 1 × 10^2^ CFU group died out from days 1 to 15 postinfection. For 1 × 10^2^ CFU group, 92% of animals survived until 12 days postinfection for necropsy.

**Figure 2:**
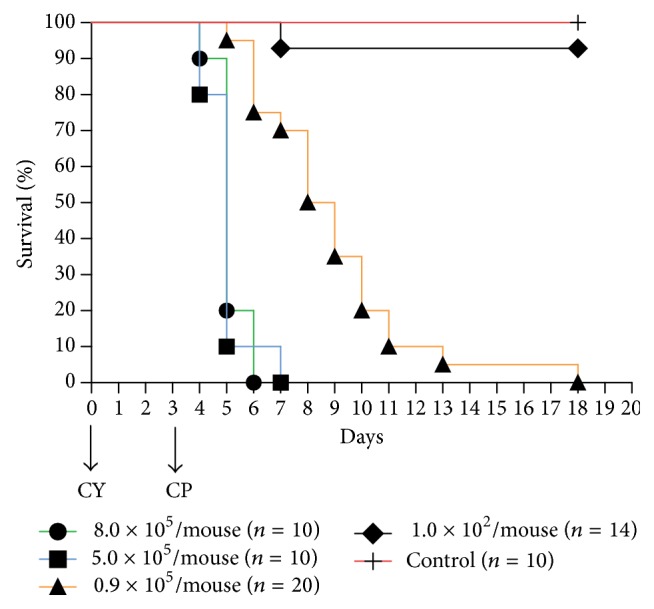
Survival curves. The mortality showed positive associations with infectious dose. And the optimal inoculum for* C. parapsilosis* was 0.9 × 10^5^ CFU per immunosuppressed mouse.

The survival curves were shown in Figure 2. 0.9 × 10^5^ CFU group showed significant differences with the other groups (*Log-rank Test*, *P* < 0.05). The median survival times of 0.9 × 10^5^ CFU, 5.0 × 10^5^ CFU, and 8.0 ×10^5^ CFU groups are 5.5 days, 2 days, and 2.5 days, respectively. These data indicated that the optimal inoculum for* C. parapsilosis *was 0.9 × 10^5^ CFU per mouse in immunosuppressed mice.

### 3.3. Clinical Signs of the Model

The clinical symptoms of distress, such as that decreased movement, decreased food and water consumption, weight loss, self-imposed isolation, and difficult breathing, were conspicuous in most infected animals 2 days postinfection. Each infected group presented death of animals during the infection period. Before death, most of them exhibited severe neurologic disorders, including opisthotonus, torticollis, and ataxia.

### 3.4. Gross Observation of the Model

**Figure 3:**
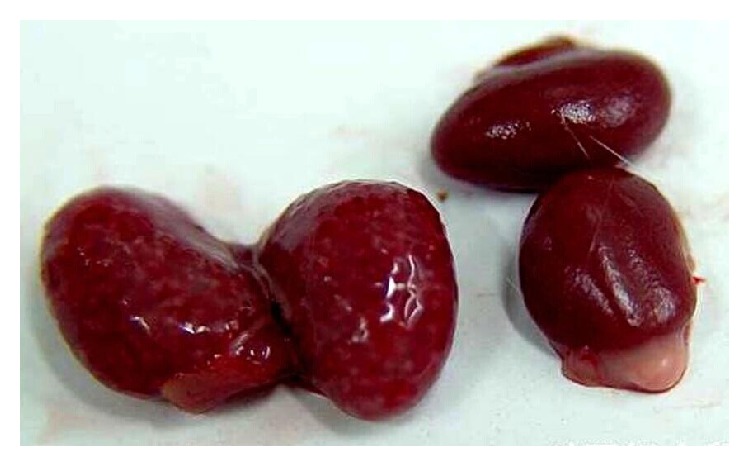
Gross observation of kidneys. The right two kidneys were the normal controls and the left swollen kidneys covered with petechiae were from infected mice.

At necropsy, swollen kidneys covered with white foci were observed in all infected mice (Figure 3). Some cases showed petechiae and petechial bleeding on the surface of the lung lobes and brains. Besides, moderate enlargements of the spleens were observed in most animals. No specific gross lesions were found in other organs.

### 3.5. Histological Analysis of the Model

At day 1, the lesions in all organs were relatively temperate, characterized by inflammation and slightly fungal infiltration of kidney tissues.

**Figure 4:**
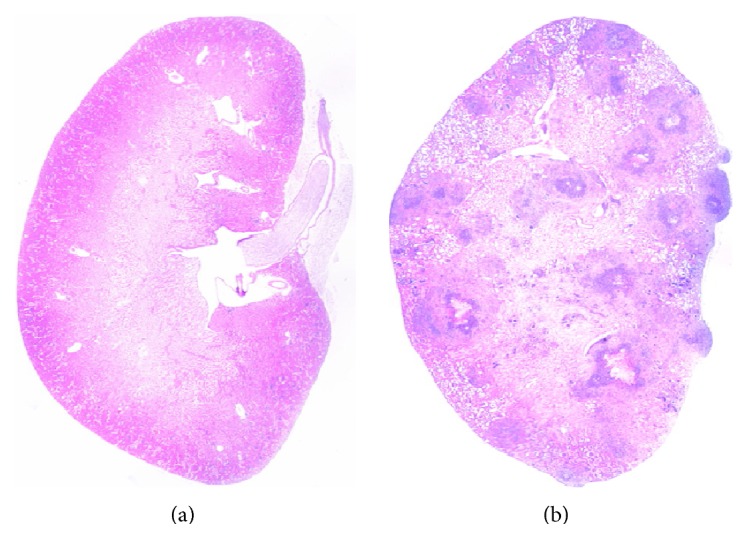
Histopathological changes in kidneys.  Figure 4(a) was the control mouse showing no observed lesions.  Figure 4(b) was the infected mouse presenting numerous granulomas in tissue section of the kidney.

**Figure 5:**
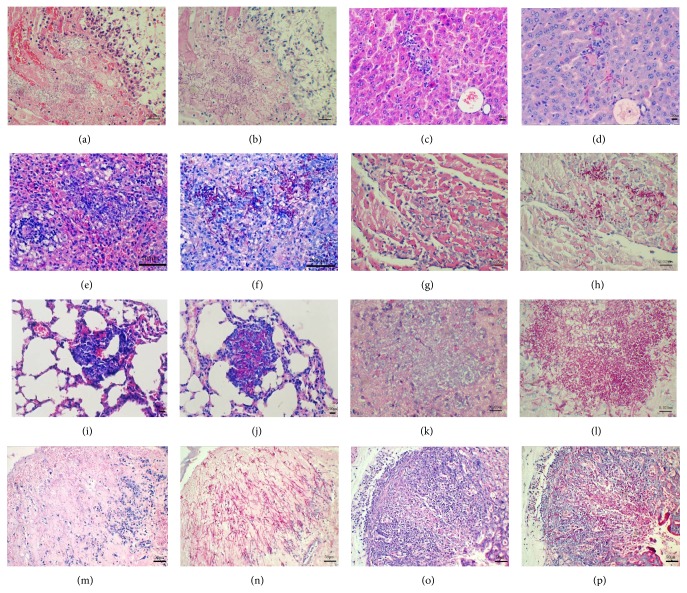
Histological findings of infected immunosuppressed mice. Figures 5(a), (c), (e), (g), (i), (k), (m), and (o) were H&E stained sections displaying the representative lesions in kidney, liver, spleen, heart, lung, brain, stomach, and bladder walls, respectively. Figures 5(b), (d), (f), (h), (j), (l), (n), and (p) stained by PAS showed fungal mycelia infiltration in kidney, liver, spleen, heart, lung, brain, stomach, and bladder walls, respectively.

At s 4 and 6, representative lesions could be observed in most organs. The kidney suffered the most severe lesions compared to the other organs or tissues. Numerous granulomas were diffused in renal cortex and medulla (Figure 4). Microscopic examination revealed that all granulomas contained fungal mycelia, blastospores, and chlamydospore-like structures (Figures 5(a) and 5(b)). The liver only experienced minor lesions, presenting periportal infiltration with a few polymorphonuclear leukocytes and fungal elements (Figures 5(c) and 5(d)). The spleen demonstrated the nonspecific lesion of the decrease of lymphocytes in white pulp and infiltration of a few PAS-positive fungal elements (Figures 5(e) and 5(f)). A certain number of granulomas or focal necrosis could be observed in the cardiac muscles, which were always composed by a necrotic center, infiltration of leukocytes, mycelia, blastospores, and PAS-positive components (Figures 5(g) and 5(h)). Some certain focal necroses infiltrated by erythrocytes, lymphocyte-like cells, PAS-positive mycelia, blastospores, and chlamydospore-like structures were found in lung interstitium (Figures 5(i) and 5(j)). The lesions in brains were relatively more severe than the other organs, except kidneys. Focal liquefactive necrosis with abundant invading fungal pseudohyphae disrupted much of the forebrain (Figures 5(k) and 5(l)). Multifocal to coalescent necrosis and fungal invasions were observed in the gastric wall, which destroyed the deep structures of gastric wall including lamina propria, lamina muscularis, and stratum subvascular (Figures 5(m) and 5(n)). Similar lesions and extensive fungal invasion to gastric wall were also present within the bladder wall (Figures 5(o) and 5(p)).

### 3.6. Tissue Fungal Burdens of the Model

**Figure 6:**
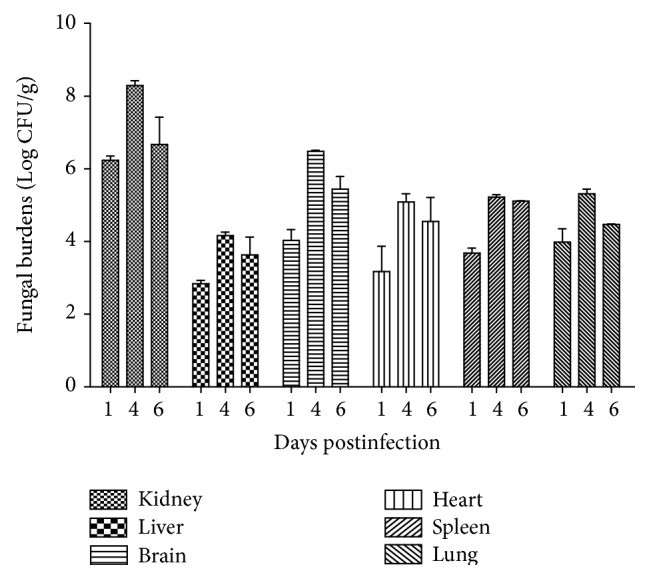
Dynamic changes of tissue fungal burdens. Tissue fungal burdens showed a transient increase during the infection period. And the kidney suffered the highest CFU scores.

*C. parapsilosis* could be detected out from the kidney, liver, brain, heart, spleen, and lung tissue suspension from day 1 postinfection. The dynamic changes of fungal burdens were the same in these organs, showing a transient increase with a brief peak at day 4 postinfection (Figure 6). The kidneys presented with much higher CFU scores compared with the other organs of the same time (*t*-test, *P* < 0.05).

### 3.7. Cytokine Levels of the Model

**Figure 7:**
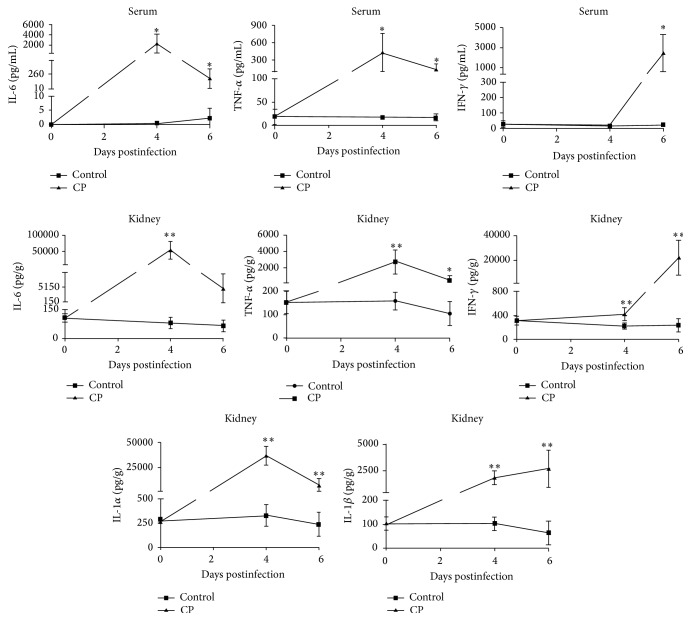
Dynamic changes of serum and renal cytokines. Serum IL-6, TNF-*α*, and IFN-*γ* and renal IL-6, TNF-*α*, IFN-*γ*, IL-1*α*, and IL-1*β* presented significant increases during the infection period.

For the CY group, all the detected serum and renal cytokines showed no significant changes all the time. Unlike the CY group, the* C. parapsilosis* infected mice presented different dynamic changes in serum and renal cytokines (Figure 7). Serum IL-6 and TNF-*α* showed a transient increase and reached the highest points at day 4, followed by the increase of serum IFN-*γ*. The other serum cytokines (IL-2, IL-4, IL-5, IL-10, IL-17, IL-23, GM-CSF, IL-1*α*, and IL-1*β*) showed no specific changes. Renal IL-6, TNF-*α*, IFN-*γ*, IL-1*α*, and IL-1*β* presented significant increases from days 4 to 6 postinfection. However, the other renal cytokines (IL-2, IL-4, IL-5, IL-10, IL-17, IL-23, and GM-CSF) presented no specific changes.

### 3.8. Model Based Evaluation of Antifungal Activity of Fluconazole

**Figure 8:**
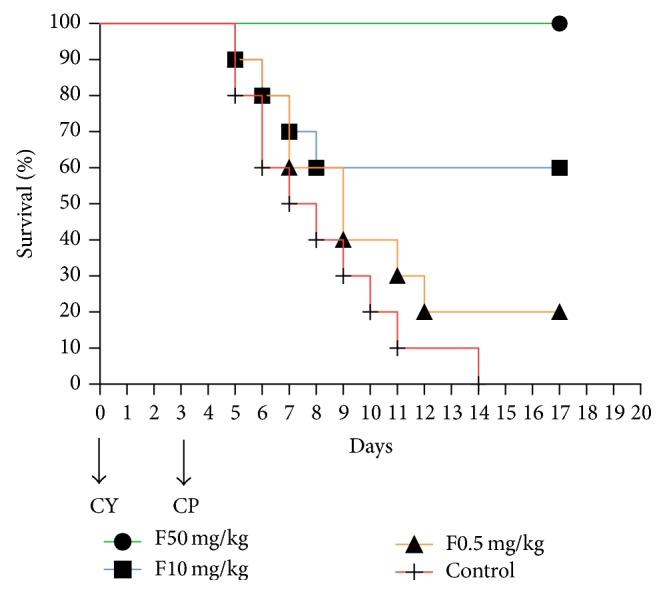
Survival curves of fluconazole treated mice (*n* = 10 per group). Comparisons between each two groups except 0.5 and 10 mg/kg/d groups that showed significant differences (*Log-rank Test, P* < 0.05).

**Figure 9:**
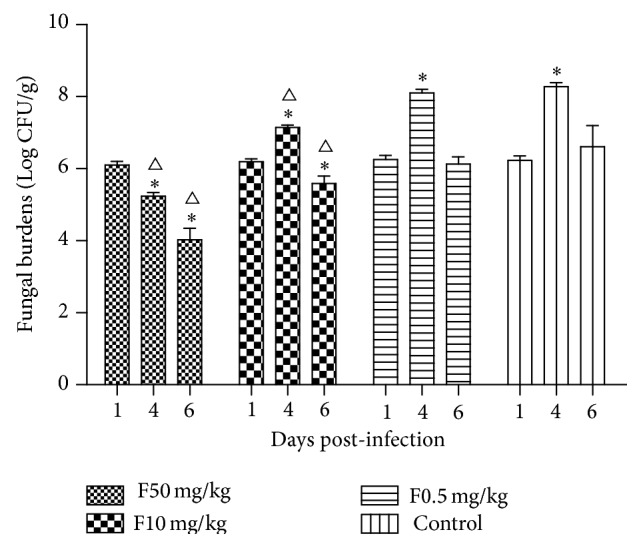
Renal fungal burdens of fluconazole treated mice (*n* = 5 per day). 50 mg/kg/d group showed a significant decline of renal fungal burdens, while, the other groups showed a transient increase during the experiment period. _ _^*∗*^ *P* < 0.05, *t*-test, versus that of day 1. _ _^∆^ *P* < 0.05, *t*-test, versus that of control of the same time point.

Four groups of infectious models were used to evaluate the antifungal efficiency of fluconazole by administrating different dosages of fluconazole. During the experiment period, all animals of 50 mg/kg/d group survived, while mortalities of 100%, 80%, and 40% were observed in 0, 0.5, and 10 mg/kg/d groups, respectively. Survival curves were generated and compared between each 2 groups (Figure 8). Significant differences were found between 50 mg/kg/d group and the other groups (*Log-rank Test*, *P* < 0.05). There were also significant differences between 10 mg/kg/d group and the others. No significant differences were shown between 0.5 mg/kg/d group and the control. After receiving fluconazole, 50 mg/kg/d group showed a significant decline of renal fungal burdens (Figure 9). Though there were declines in mortalities of 0.5 and 10 mg/kg/d groups, the renal fungal burdens significantly increased at day 4 postinfection (*t*-test, *P* < 0.05) (Figure 9). When compared with the control, renal fungal burdens of both 50 and 10 mg/kg/d groups at days 4 and 6 were significantly lower than the control (*t*-test, *P* < 0.05). Results indicated that there were ideal antifungal activities for fluconazole at dosages of 10–50 mg/kg/d.

## 4. Discussion

*C. albicans *has been kept as the major species associated with human* Candida *infections for decades. And the majority of experimental animal models for* Candida* species have focused on* C. albicans* too. Though many kinds of animals have been used to study* Candida *infections, the rodent infection models occupied the majority for economic reasons, easy handling, and the availability of genetic modification [10]. In this paper, we will try to introduce an ideal* C. parapsilosis* infection model based on immunosuppressed ICR mice.

Many regents have been used to obtain the immunosuppressed mice, for example, cortisone acetate and hydrocortisone succinate [11]. Here, we used CY to induce immunosuppression in ICR mice. Mice presented a time transient decrease of the total leukocyte counts and reached the lowest point at day 4 after receiving CY for 3 consecutive days at a dosage of 100 mg/kg body weight. Result demonstrated that days 3 to 6 after CY administration were the optimal infection time points. And the day 4 was chosen to infect mice in this study.

To assure the infectious dose of* C. parapsilosis,* immunosuppressed mice were infected with 4 dosages, respectively. According to the results of survival curves, the dosage of 0.9 × 10^5^ CFU* C. parapsilosis *was the optimal infectious dose in immunosuppressed ICR mice. The morphology of* Candida* species was important to animal models. Our previous experiments showed that mycelium* C. parapsilosis* has more virulence than fungal spores. And the inoculums containing >95% fungal spores were used in this study.

The infection model based on 0.9 × 10^5^ CFU* C. parapsilosis* presented a median survival time of 5.5 days, which was long enough to satisfy researches on antifungal agents. Histopathology and tissues fungal burdens revealed the model to be a systemic infection one, and the kidneys suffered the most severe lesions, indicating that kidney could be a suitable target organ for screening antifungal agents. The fungal burdens showed a transient increase with a brief peak at day 4 postinfection in multiple organs, demonstrating that day 4 postinfection was an optimal time point to assess the fungal burdens for research or evaluation of antifungal agents, which were further identified by using the infectious model in evaluation of antifungal activity of fluconazole. Some serum and renal proinflammatory cytokines were induced and consistent with the tissue fungal burdens, while anti-inflammatory cytokines showed no significant increase. Th1/Th2 cytokine imbalance might result in high mortality of infected mice.

This project established a systemic* C. parapsilosis* infection model in immunosuppressed ICR mice. In this model, multiorgans, including kidneys, liver, lungs, spleen, brains, and bladder wall, represented numerous microabscesses, which demonstrated congestion, hemorrhages, tubular degeneration, and heterophilic infiltration. This model affords opportunities for increasing our understanding of fungal pathogenesis and treatment.
